# Bovine Viral Diarrhea Virus (BVDV): A Preliminary Study on Antiviral Properties of Some Aromatic and Medicinal Plants

**DOI:** 10.3390/pathogens10040403

**Published:** 2021-03-29

**Authors:** Silvia Madeddu, Alessandra Marongiu, Giuseppina Sanna, Carla Zannella, Danilo Falconieri, Silvia Porcedda, Aldo Manzin, Alessandra Piras

**Affiliations:** 1 Department of Biomedical Sciences, University of Cagliari, Cittadella Universitaria, 09042 Monserrato, Italy; silvia.madeddu@unica.it (S.M.); a.marongiu24@studenti.uniss.it (A.M.); aldomanzin@unica.it (A.M.); 2 Department of Biomedical Sciences, University of Sassari, 07100 Sassari, Italy; 3 Department of Experimental Medicine, University of Study of Campania “Luigi Vanvitelli”, 80138 Naples, Italy; carla.zannella@unicampania.it; 4 State Industrial Technical Institute “Michele Giua”, 09100 Cagliari, Italy; danilo.falconieri@tiscali.it; 5 Department of Chemical and Geological Sciences, University of Cagliari, Cittadella Universitaria, 09042 Monserrato, Italy; porcedda@unica.it

**Keywords:** BVDV, viral infections, antiviral activity, essential oils, α-humulene

## Abstract

Plant products provide an alternative and successful source of lead compounds for the pharmaceutical industry. The present study was aimed to evaluate, in cell-based assays, the antiviral properties of essential oils obtained from plants that commonly grow in Sardinia, Italy, against a broad spectrum of RNA/DNA viruses. The essential oils of *Helichrisumitalicum* (Roth) G. Don ssp. *microphyllum* (Willd.) Nyman, *Laurus nobilis* L., *Mirtuscommunis* L., *Pistacia lentiscus* L., *Salvia officinalis* L., *Saturejathymbra* L., *Lavandula angustifolia* Mill., *Foeniculum vulgare* Mill., and *Eucalyptus globulus* Labill. were extracted by hydrodistillation and analyzed by gas chromatography mass spectrometry (GC–MS). Interestingly, the essential oil of *Salvia officinalis* showed moderate activity against bovine viral diarrhea virus (BVDV), an enveloped RNA virus belonging to the *Flaviviridae* family. BVDV is responsible for several clinical manifestations in bovines, including respiratory, gastroenteric, and reproductive diseases, with a significant economic impact. With the aim to individuate the constituent of the *Salvia officinalis* responsible for the biological activity, we tested the major components of the oil: camphene, β-pinene, limonene, 1,8-cineole, cis-thujone, camphor, (E)-caryophyllene, and α-humulene. Here, we describe α-humulene as an active component that is non-cytotoxic and active against BVDV (EC_50_ = 36 µM). Its antiviral effects were evaluated using virucidal cytopathic effect inhibition and viral yield reduction assays. This is the first scientific report showing the anti BVDV effects of *Salvia officinalis* essential oil and α-humulene as the main active component.

## 1. Introduction

Bovine viral diarrhea virus (BVDV) is a small, enveloped virus with a single-stranded, positive-sense RNA genome belonging to the genus Pestivirus in the *Flaviviridae* family. BVDV is the etiological agent of serious diseases in cattle and other ruminants [1,2,3]. Infection by BVDV induces a range of clinical manifestations including respiratory problems, gastroenteric diseases, and reproductive diseases. There are two biotypes: cytopathic (CP) and non-cytopathic (NCP). CPs are related to mucosal diseases with ulceration of the gastrointestinal tract, diarrhea, and dehydration up to the death of the subject, while the NCP biotype is related to persistent infection (PI) [4,5]. Animals exhibiting PI remain viremic for life and serve as persistent reservoirs of viruses [6]. PI by BVDV in a pregnant cow can result in a wide range of clinical manifestations, such as resorption; abortion; mummification; or in case of fetuses who survive early infection, may be abnormally formed, blind, or with skeletal defects, weak immune system, respiratory problems, or have an underdeveloped brain [7,8].

All of these clinical manifestations, in both males and females, result in a high mortality rate in cattle worldwide, causing extensive economic damage due to loss of milk production, reproductive waste, and enhanced risk of morbidity and mortality [9,10].

Despite the infections ofthis pathogen havingthe highest impact on livestock worldwide [1,11], it is still the case that neither effective vaccines nor selective antiviral drugs are available [12,13].

The search for potential anti-BVDV agents has led to the exploration of both natural substances and synthetic compounds [14,15,16], leading to the choice of natural substances rather than nucleoside analogs that are capable of causing negative side effects once administered [17].

Natural products derived from plants represent an important source for the pharmaceutical industry and are currently being investigated to find new pharmacological strategies. Compounds originated from plants and plant extracts have huge potential and are characterized by higher biocompatibility and safety.

The present study was conducted to evaluate the in vitroantiviral activities of the essential oils obtained from plants collected in Sardinia, Italy. Essential oils of *Helichrisumitalicum*(Roth) G. Don ssp. *microphyllum* (Willd.) Nyman, *Laurus nobilis* L., *Mirtuscommunis* L., *Pistacia lentiscus* L., *Salvia officinalis* L., *Saturejathymbra* L., *Lavandula angustifolia* Mill., *Foeniculum vulgare* Mill., and *Eucalyptus globulus* Labill. were tested in cell-based assays against a broad spectrum of RNA/DNA viruses.

The essential oil of *Salvia officinalis* showed moderate activity (EC_50_ of 50 ± 3 µg/mL) against BVDV. Among the various constituents of the essential oil of *Salvia officinalis L.*, the trans-caryophylleneshowed slight activity (EC50 of 90 µM) and the monocyclic sesquiterpene α-humulene was found as an active component (EC50 of 36 µM).

## 2. Results and Discussion 

The study described in this paper investigatedthe antiviral effect of essential oils obtained from plants that commonly grow in Sardinia against a broad spectrum of viruses.

Essential oils of *Helichrisumitalicum* (Roth) G. Don ssp. microphyllum (Willd.) Nyman, *Laurus nobilis* L., *Mirtuscommunis* L., *Pistacia lentiscus* L., *Salvia officinalis* L., *Saturejathymbra* L., *Lavandula angustifolia Mill*., *Foeniculum vulgare Mill*., and *Eucalyptus globulus Labill*.were evaluated for their potential antiviral activity.

**Figure 1:**
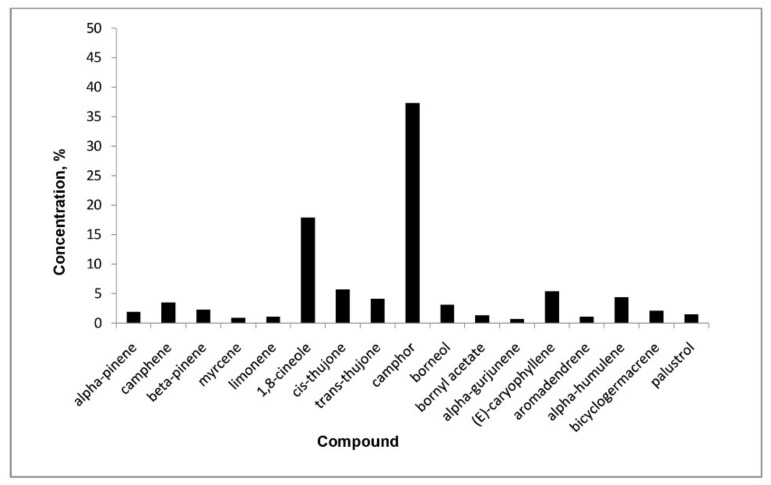
Chemical compositions of the essential oil (EO) from *Salvia officinalis* (main components).

As shown in Table S1, there were no cytotoxic effects on cell lines evaluated in 3-(4,5-dimethylthiazol-2-yl)-2,5-diphenyltetrazolium bromide (MTT) assay (CC_50_ > 100 µg/mL). The essential oils investigated did not show antiviral activity against HIV-1 or representatives of Negative-strand RNA viruses (ss-RNA)-viruses (hRSV, VSV) with an EC_50_ > 100 µg/mL. In the group of viruses chosen as representative of ss-RNA^+^ viruses (DENV-2, WNV, YFV, CV-B5, poliovirus Sb-1), essential oils were not endowed with great effects, with the only exception being on BVDV. Indeed, *Salvia officinalis* L. was shown to have moderate antiviral activity on BVDV (EC_50_ = 50 ± 3 µg/mL). The essential oil was characterized, and the major constituents are shown in Figure 1. 

Once the active essential oil was identified, we explored it for anti-BVDV activity and its main constituents; the results are reported in Table 1.

**Figure 2:**
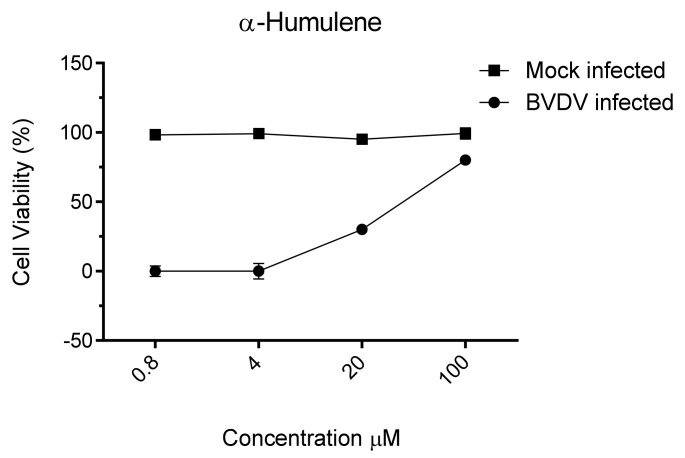
Cytotoxicity and anti BVDV activity of α-humulene. The viability of BVDV-infected MDBK cells was estimated by MTT assay, threedays after-infection. The number of live cells was expressed as a percentage of mock-infected, untreated control cells. Data are expressed as means ± SD of at least three independent measurements.

No component tested on the MDBK cell line showed toxic effects at the concentrations evaluated. *Trans-E-caryophyllene* and *α-humulene* resulted in the only constituents endowed with antiviral activity, showing EC_50_ values of 90 µM and 36 µM, respectively. These results prompted us to investigate *α-humulene* as the main active component (Figure 2).

A virucidal assay against BVDV virions was carried out in order to investigate the possibility that α-humulene acts directly on the viral particle, leading to infectivity inactivation. 

**Figure 3:**
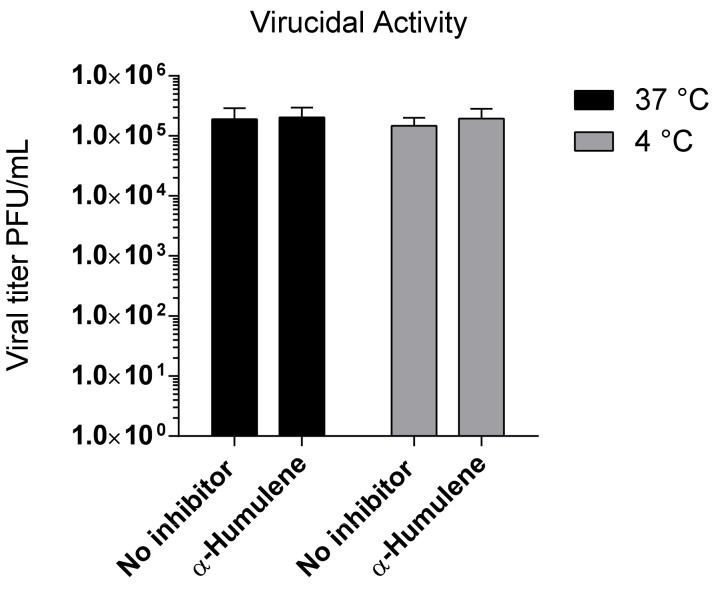
Virucidal activity of α-humulene. BVDV suspensions were incubated with title compound (100 µM) at 4 and 37 °C for 2 h, and then the remaining infectivity was determined. Results are expressed as titer reduction in compound-treatedsamples compared to non-treated samples. Each value represents the mean of duplicate assays ± standard deviation.

No significant differences were observed between the titer of BVDV treated for 2 h with the compound and the untreated controls at the two different temperatures of 4 and 37 °C, as shown in Figure 3. Results indicate that the inhibitory effect detected by the antiviral assay could be due to interference with a phase along the BVDV replication cycle.

**Figure 4:**
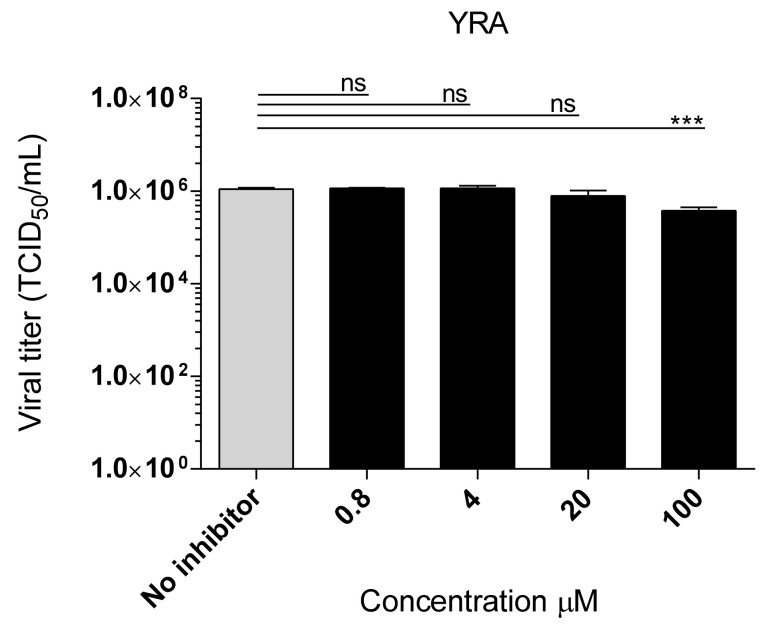
The yield of infectious BVDV virus produced in infected MDBK cells treated with α-humulene. MDBK cells were infected with BVDV (multiplicity of infection, m.o.i. = 0.1). The infected cultures were treated with α-humulene at indicated doses (100, 20, 4, 0.8 µM). Viral yields in the culture supernatant were collected and tittered 72 h after infection. Results are expressed as mean ± standard deviationfrom threeseparate experiments done in triplicate. Statistically significant differences are expressed as follows: *** = *p* < 0.001, ns =not statistically significant.

For this reason, the antiviral activity of α-humulene was analyzed in a yield reduction assay in order to establish the reduction of BVDV titer in the presence of the active compound during a single round of BVDV infection. Non-cytotoxic concentrations of 100, 20, 4, and 0.8 µM were used, and a dose-dependent reduction of virus titer was observed (Figure 4).

*Trans-E-caryophyllene*, also known as β-*caryophyllene* and *α-humulene,* are sesquiterpenes, the main active components of essential oils in the plant kingdom. Different research groups described their biological properties as having analgesic, antitumor, and antioxidant effects [18,19,20]. Furthermore, they can work synergistically with conventional chemotherapeutic agents thanks to theirstrong pain-relieving action [21,22,23]. They are the main components of several essential oils that have been found to beactive against bacteria, fungi, and protozoa [24,25,26].

Currently, although many essential oils containing β-caryophyllene and α-humulene have been reported as endowed with antiviral activities [27,28,29,30,31,32,33], α-humulene has not been described as a specific active component.

Furthermore, several studies have shown that essential oils from *Ocimum basilicum*, *Lippia graveolens,* and *Atalantia sessiflora Guillaumin*, characterized by many active components [34,35,36], were found to beactive against BVDV, but their mechanisms of action are not still clear.

Interestingly, Venturi et al. described a remarkable anti HSV-1 activity of the essential oil from *Glechon spathulata* and *Glechon marifolia*. *Glechon marifolia* oil’s main components are α-humulene at 23.3 % and β-caryophyllene at 32 %. The authors hypothesized that α-humulene could act by interfering at a later stage of the replicative cycle of the virus inside the cell [27].

In this paper, we did not investigate the activities of each component, and therefore it could be the case that α-humulene and β-caryophyllene could exert antiviral effects as a result of the complex interactions between all the constituents or alone.

## 3. Materials and Methods

### 3.1. Plant Material and Essential Oil Isolation 

The leaves of samples were collected in Sardinia between March and October of 2010 by Dr. Alessandra Piras. A voucher specimen of the plants is kept at the Department of Chemical and Geological Sciences, University of Cagliari (Italy). Before utilization, plant materials were air-dried and were ground with a Malavasi mill (Bologna, Italy). Isolation of essential oils by hydrodistillation was performed in a Clevenger-type apparatus for 4 h [37]. The essential oils were stored at 4 °C in the dark until the chemical analyses.

### 3.2. Essential Oil Analysis

Analyses of essential oils were carried out in a gas chromatograph equipped with a flame ionization detector (FID) and in a gas chromatograph fitted with a quadrupole mass spectrometer, as previously reported [38].

### 3.3. Cells and Viruses

All cell lines were obtained from American Type Culture Collection (ATCC): CD4+ human T-cells (MT-4), Madin-Darby bovine kidney (MDBK) (ATCC CCL 22 (NBL- 1) *Bos taurus)*, baby hamster kidney (BHK-21) (ATCC CCL 10 (C-13) *Mesocricetus auratus*), and monkey kidney (Vero-76) (ATCC CRL 1587 *Cercopithecus aethiops*). 

Human immunodeficiency virus type-1 (HIV-1) IIIB laboratory strain was derived from supernatant of H9/IIIB cells (NIH 1983). Viruses representative of ssRNA+, of ssRNA-, and of DNA virus were, respectively, (i) yellow fever virus (YFV) (strain 17-D vaccine (Stamaril Pasteur J07B01)), bovine viral diarrhea virus (BVDV) (strain NADL (ATCC VR-534)), west Nile virus (WNV) (clinical isolate), dengue virus (DENV-2) (clinical isolate), coxsackie type B5 (CV-B5), strain Faulkner (ATCC VR-185), and poliovirus type-1 (Sb-1), Sabin strain Chat (ATCC VR-1562); (ii) human respiratory syncytial virus (hRSV) (strain A2 (ATCC VR-1540)) and vesicular stomatitis virus (VSV) (lab strain Indiana (ATCC VR 1540)); and (iii) vaccinia virus (VV) (vaccine strain Elstree-Lister (ATCC VR-1549)) and human herpes 1 (HSV-1) (strain KOS (ATCC VR- 1493)). 

### 3.4. Cytotoxicity Assays 

Cytotoxicity tests were run in parallel with their antiviral activity through the viability of mock-infected treated cells, as monitored by the MTT method [39] and as previously described [33].

### 3.5. Antiviral Assays

Compounds’ activity against HIV-1 was determined by the MTT method, as reported previously [34]. Compounds’ activity against DENV-2, WNV, and YFV was based on inhibition of cytopathogenicity virus-induced in BHK-21 cells acutely infected at an m.o.i. of 0.01. Compounds’ activity against BVDV was based on inhibition of virus-induced cytopathogenicity in MDBK cells acutely infected at an m.o.i. of 0.01. Briefly, BHK and MDBK cells wereplated in 96-well plates (5 × 10^4^ and 3 × 10^4^ cells per well, respectively). The monolayers were then infected with a proper virus dilution (50 μL) of MEM-Earl with L-glutamine, 1 mM sodium pyruvate, and 0.025 g/L kanamycin, with 1% inactivated Fetal Bovine Serum (FBS). Moreover, 50 μL of medium, with or without serial dilutions of test compounds, was added. After 72 h of incubation at 37 °C, cell viability was determined by the MTT method.

Compounds’ activity against CV-B5, Sb-1, VSV, VV, HSV-1, and RSV was determined by plaque reduction assays in infected cell monolayers, as described previously [39]. 

The extent of cell viability and viral replication, at each sample concentration evaluated, were expressed as a percentage of non-treated controls.

Concentrations leading to 50% inhibition (CC_50_ or EC_50_) were calculated by linear regression analysis.

### 3.6. Virucidal Activity 

A suspension of viral particles was directly exposed to the test compound and incubated for 2 h at 37 and 4 °C. Then, these mixtures were serially diluted, and infectious titers were compared with those obtained with non-treated viral suspension. 

### 3.7. Yield Reduction Assay

MDBK cells wereplated in 24-well plates (6 × 10^5^ cells per well). Subsequently, the BVDV inoculum was prepared at an m.o.i. of 0.1 and incubated at 37 °C for 1 h. Then, the virus-containing medium was replaced with a minimum essential medium (E-MEM) supplemented with 1% of horse serum and the compound. After 72 h, supernatants were collected and TCID_50_ titers were quantified by the Reed and Muench endpoint titration method.

## 4. Conclusions

In this study, we described the anti-BVDV activity of *Salvia officinalis* essential oil and itsactive components, *α*-humulene and β-caryophyllene. 

In particular, *α*-humulene was found to be non-cytotoxic and was able to reduce the viral BVDV titer in vitro assays in a dose-dependent manner. These results require further investigation and additional studies to understand the mechanism of action of the active molecule. Although they have been shown to have interesting potential against BVDV, the constituent mixture of *Salvia officinalis* Essential Oil has no greater potential than a single constituent. To the best of our knowledge, this is the first scientific communication reporting the anti-BVDV effects of *Salvia officinalis* essential oil with α-humulene as the main active component.

## Figures and Tables

**Table 1:** Cytotoxicity and anti-bovine viral diarrhea virus (BVDV) activity of *Salvia officinalis* essential oil and their main constituents.

Compounds	MDBK	BVDV
	^a^ CC_50_	^b^ EC_50_
* Salvia officinalis	> 100	50 ± 3
Camphor	> 100	> 100
Camphene	> 100	> 100
2+β Thujone	> 100	> 100
Limonene	> 100	> 100
β-Pinene	> 100	> 100
Trans-Caryophyllene	> 100	90 ± 1
α-Humulene	> 100	36 ± 0.5
1,8-Cineole	> 100	> 100
Reference Compound		
2′-C-Me-Guanosine	> 100	1.6 ± 0.2

^a^ Compound concentration (μM) required to reduce the viability of mock-infected Madin-Darby bovine kidney (MDBK) cells by 50%, as determined by the MTT method. ^b^ Compound concentration (μM) required to achieve 50% protection of MDBK cells from the BVDV-induced cytopathogeneticy, as determined by the MTT method. * (µg/mL). Data are mean values + standard deviation (SD) for three independent determinations. When SD is not shown, the variation among samples results in less than 15%. CC_50_ (50% cytotoxic concentration), EC_50_ (50% effective concentration).

## Data Availability

Data sharing not applicable.

## Supplementary Materials

The following are available online at https://www.mdpi.com/article/10.3390/pathogens10040403/s1: Table S1: Cytotoxicity and antiviral activity of essential oils and reference compounds against representatives of ssRNA^+^ (HIV-1, BVDV, DENV-2, WNV, YFV, CV-B5, Sb-1), ssRNA^-^ (RSV, VSV), and double-stranded DNA viruses dsDNA (VV, HSV-1) viruses.
