# Supplementary material for: Bovine Viral Diarrhea Virus (BVDV): A Preliminary Study on Antiviral Properties of Some Aromatic and Medicinal Plants
Source: Pathogens. 2021 Mar 29;10(4):403. doi: 10.3390/pathogens10040403 (PMC8066157; doi:10.3390/pathogens10040403)
Supplement: Supplementary file 1 [file pathogens-10-00403-s001.pdf]

Table S1. Cytotoxicity and antiviral activity of Essential oils and reference compounds against representatives of ssRNA^{+} (HIV-1, BVDV, DENV-2, WNV, YFV, CV-B5, Sb-1), ssRNA^{-} (RSV, VSV), and dsDNA (VV, HSV-1) viruses.

**Compds**	**MT-4**	**HIV-1**	**MDBK**	**BVDV**	**BHK**	**DENV -2**	**WNV**	**YFV**	**Vero76**	**CV-B5**	**Sb-1**	**RSV**	**VSV**	**VV**	**HSV-1**
	$^{a}CC_{50}$	$^{b}EC_{50}$	$^{c}CC_{50}$	$^{d}EC_{50}$	$^{e}CC_{50}$	$^{f}EC_{50}$	$^{g}EC_{50}$	$^{h}EC_{50}$	$^{i}CC_{50}$	$^{j}EC_{50}$	$^{k}EC_{50}$	$^{l}EC_{50}$	$^{m}EC_{50}$	$^{n}EC_{50}$	$^{o}EC_{50}$
Lavandula angustifolia	>100	>100	>100	>100	>100	>100	>100	>100	>100	>100	>100	>100	>100	>100	>100
Foeniculum vulgare	>100	>100	>100	>100	>100	>100	>100	>100	>100	>100	**100**	>100	>100	>100	>100
Salvia officinalis	>100	>100	>100	**50±3**	>100	>100	>100	>100	>100	>100	>100	>100	>100	>100	>100
Myrtus communis	>100	>100	>100	>100	>100	>100	>100	>100	>100	>100	>100	>100	>100	>100	>100
Pistacia lentiscus	>100	>100	>100	>100	>100	>100	>100	>100	>100	>100	>100	>100	>100	>100	>100
Eucalyptus globulus	>100	>100	>100	>100	>100	>100	>100	>100	>100	>100	>100	>100	>100	>100	>100
Laurus nobilis	>100	>100	>100	>100	>100	>100	>100	>100	>100	>100	>100	>100	>100	>100	>100
Helichrysum italicum ssp. microphyllum	>100	>100	>100	>100	>100	>100	>100	>100	≥100	>100	>100	>100	>100	>100	>100
Satureja thymbra	>100	>100	>100	>100	>100	>100	>100	>100	≥100	>100	>100	>100	>100	>100	>100
***Reference Compounds**															
Efavirenz	40	**0.002**													
Pleconaril									>100	0.005					
2′-C-Me-Gua			>100	**1.6**	60.0	**0.3**	**0.4**	**1.2**							
6-Aza-Uridine									4.0			1.0			
Mycophen.Ac.									20					**1.5**	
AcycloGuo															

[a] Compd concn (µg/ml) required to reduce by 50 % the proliferation of mock-infected MT-4 cells, as determined by the MTT method.
[b] Compd concn (µg/ml) required to achieve 50 % protection of MT-4 cells from HIV-1-induced cytopathogenicity, as determined by the MTT method.
[c] Compd concn (µg/ml) required to reduce by 50 % the viability of mock-infected MDBK cells, as determined by the MTT method.
[d] Compd concn (µg/ml) required to achieve 50 % protection of MDBK cells from BVDV-induced cytopathogenicity, as determined by the MTT method.
[e] Compd concn (µg/ml) required to reduce by 50 % the viability of mock-infected BHK cells, as determined by the MTT method.
[f-h] Compd concn (µg/ml) required to achieve 50 % protection of BHK cells from DENV-2[(f)]-, WNV[(g)]-, YFV[(h)]-induced cytopathogenicity, as determined by the MTT method.
[i] Compd concn (µg/ml) required to reduce by 50 % the viability of mock-infected Vero-76 cells, as determined by the MTT method.
[j-o] Compd concn (µg/ml) required to reduce by 50 % the plaque number of CV-B5[(j)], Sb-1[(k)], RSV[(l)], VSV[(m)], VV[(n)], HSV-1[(o)] in Vero-76 cells.
*Reference Compounds (µM)
